# Spatio-Temporal Fractal Dimension Analysis from Resting State EEG Signals in Parkinson’s Disease

**DOI:** 10.3390/e25071017

**Published:** 2023-07-02

**Authors:** Juan Ruiz de Miras, Chiara-Camilla Derchi, Tiziana Atzori, Alice Mazza, Pietro Arcuri, Anna Salvatore, Jorge Navarro, Francesca Lea Saibene, Mario Meloni, Angela Comanducci

**Affiliations:** 1 Software Engineering Department, University of Granada, 18071 Granada, Spain; 2 IRCCS Fondazione Don Carlo Gnocchi, 20148 Milan, Italy; 3 Department of Engineering, Università Campus Bio-Medico di Roma, 00128 Rome, Italy

**Keywords:** fractal dimension, box-counting, EEG, Parkinson’s disease, neurodegeneration

## Abstract

Complexity analysis of electroencephalogram (EEG) signals has emerged as a valuable tool for characterizing Parkinson’s disease (PD). Fractal dimension (FD) is a widely employed method for measuring the complexity of shapes with many applications in neurodegenerative disorders. Nevertheless, very little is known on the fractal characteristics of EEG in PD measured by FD. In this study we performed a spatio-temporal analysis of EEG in PD using FD in four dimensions (4DFD). We analyzed 42 resting-state EEG recordings comprising two groups: 27 PD patients without dementia and 15 healthy control subjects (HC). From the original resting-state EEG we derived the cortical activations defined by a source reconstruction at each time sample, generating point clouds in three dimensions. Then, a sliding window of one second (the fourth dimension) was used to compute the value of 4DFD by means of the box-counting algorithm. Our results showed a significantly higher value of 4DFD in the PD group (*p* < 0.001). Moreover, as a diagnostic classifier of PD, 4DFD obtained an area under curve value of 0.97 for a receiver operating characteristic curve analysis. These results suggest that 4DFD could be a promising method for characterizing the specific changes in the brain dynamics associated with PD.

## 1. Introduction

The fractal dimension (FD) [1] is a quantitative measure of shape complexity with a large number of applications in characterizing neurodegenerative diseases from both medical imaging data [2] and electroencephalogram (EEG) signals [3]. Analyzing the complexity of EEG signals is crucial for understanding the underlying brain dynamics and detecting early signs of neurodegeneration. Correlation dimension [4] and, in particular, Higuchi’s fractal dimension [5] are the most widely-used methods for computing the fractal dimension of EEG signals. Nevertheless, some other non-linear metrics, such as those related to the concept of entropy [6], are also commonly used to analyze the complexity of EEG signals. In [7], a novel methodology was presented for analyzing the complexity of the spatio-temporal dynamics in the brain based on the 3D fractal dimension (3DFD) and the 4D fractal dimension (4DFD) computed from EEG. The novelty of this approach relies on the use of source reconstruction from EEG to obtain a 3D representation of cortical activations over time, from which the values of 3DFD and 4DFD were then computed.

Parkinson’s disease (PD) is a neurodegenerative disorder which affects approximately 1% of the world’s elderly population [8]. Patients with PD suffer motor dysfunction symptoms such as gait disturbance, resting tremor, and muscle rigidity, and also non-motor symptoms such as cognitive impairment and neuropsychiatric symptoms such as anxiety, depression, and apathy, which can occur even many years before the motor symptoms [9]. Therefore, an early diagnosis is of great relevance in optimizing the clinical management of the disease [10].

Previous studies have shown that non-linear measures of EEG complexity can be of relevance in characterizing early neurodegeneration processes in PD. Müller et al. [11] showed that the correlation dimension of EEG is higher for PD patients compared to healthy subjects, especially during motor task performance. When comparing the performance of linear and non-linear methods in distinguishing resting-state EEG of PD patients from healthy subjects, non-linear measures, such as entropy, had a more powerful ability to differentiate between PD and healthy subjects, with higher entropy in PD [12]. Multiscale entropy was also used in [13] for exploring the characteristics of sleep EEG in patients with PD. The analysis of multiscale entropy revealed an increased complexity of the EEG signal during non-REM (rapid eye movement) sleep in PD compared to healthy controls. Furthermore, Han and colleagues found increased entropy (wavelet packet entropy) over the global frequency domain of resting-state EEG in patients with early PD compared to healthy subjects [14]. All these previous studies revealed that using complexity measures of EEG could be a useful tool for early diagnosis of PD. However, there is limited understanding of the fractal characteristics of EEG in PD, which our study wishes to address.

To this aim, using fractal dimension we analyzed the complexity of resting-state EEG signal in PD patients without dementia. As described above, most previous work in this topic has analyzed EEG using several measures of entropy, but very little is known about the fractal characteristics of EEG in PD. In our study, we followed a similar approach to the one presented in [7], adapted for the case of resting-state EEG, analyzing the spatio-temporal complexity in terms of the 4DFD of brain activations. The results demonstrated that patients with PD without dementia showed higher complexity values compared to the elderly, supporting the hypothesis that PD is characterized by a significant alteration in brain complexity and overall changes to the underlying organization of the brain. These findings suggest that 4DFD could be a valuable complexity measure for identifying early neurodegeneration in PD, even before it is captured by clinical scales for cognitive decline, with potential implications for enhancing early diagnosis and informing clinical practice in order to plan rehabilitation intervention.

## 2. Materials and Methods

### 2.1. Subjects

A total of 42 subjects participated in the study: 27 patients diagnosed with Parkinson’s disease (PD group) (12 females and 15 males, with a mean age of 69.59 ± 6.74), and 15 healthy control subjects (HC group) with no symptoms or history of any neuropsychiatric disorder (7 females and 8 males, with a mean age of 67.53 ± 4.94). Demographics of both groups and clinical data of patients (see Table 1) were collected during face-to-face interviews by a neurologist and a neuropsychologist specialized in movement disorders.

For each PD patient, the disease was diagnosed on the basis of their medical history and neurological examinations. Specifically, the diagnosis of PD was made following the Movement Disorder Society (MDS) clinical diagnostic criteria [15]. All patients also underwent a global cognitive assessment including Mini-Mental Parkinson (MMP) and Montreal Cognitive Assessment (MoCA) as recommended by the MDS PD-MCI study group [16]. Exclusion criteria were: a diagnosis of Parkinson’s disease dementia (PD-D) based on MDS Task Force criteria [17], the presence of vascular Parkinsonism, drug-induced Parkinsonism, any suggestive features of a diagnosis of atypical Parkinsonism, concomitant neurologic and/or psychiatric diseases, and any other severe comorbidities which may significantly influence cognitive testing. As a global measure of the severity of motor symptoms, the MDS-Unified Parkinson’s Disease Rating Scale part III (UPDRS III) [18] was also employed. A modified Hoehn and Yahr’s scale [19] between 2 and 3 was also employed as a criterion to recruit patients with mild-to-moderate bilateral motor disability. Table 1 shows mean and standard deviation for all these scores in the PD group.

All subjects gave their informed consent for participation in the study (for details of the ethical protocol, please refer to the specific section below).

### 2.2. EEG Acquisition and Processing

During EEG recordings, both patients and healthy controls were seated in an upright or slightly reclined position in a quiet room. Spontaneous resting EEG recordings were acquired while at rest with their eyes open, not engaging in any task for a minimum of 5 min. The EEG session took place approximately 1–2 h after the administration of morning medications.

EEG was recorded with a Brainamp DC (Brain Products GmbH, Germany) equipped with 62 channels following the standard 10–20 montage. In all the recordings, reference and ground electrodes were located on the forehead and two additional channels were used to record the electrooculogram (EOG) in a diagonal montage to monitor for eye-blinking and saccades. Impedance at all electrodes was kept below 5 kOhm. Spontaneous EEG data were collected at 1000 Hz sampling rate and with a hardware filtering between 0.016 and 250 Hz. Due to the computational restrictions of further analysis, a resampling process to 500 Hz was performed.

EEG preprocessing was performed using MATLAB R2016b (Mathworks Inc., Natick, MA, USA) and custom-made scripts based on the EEGLab toolbox [20]. EEG was band-pass filtered (3rd order Butterworth, 0.5 Hz cutoffs, using the filtfilt MATLAB function) and notch-filtered (50 Hz harmonics up to 250 Hz). A trained researcher manually rejected artifactual epochs and bad electrodes. Artifactual electrodes were interpolated using spherical splines. Electrodes were then re-referenced to the average reference. Additional analysis of artifactual components through independent component analysis (ICA) decomposition allowed us to visually identify and reject components from ocular, muscular, and cardiac origin.

Source modelling was performed using the Brainstorm software [21], which is freely available for download online (http://neuroimage.usc.edu/brainstorm, accessed on 1 July 2023). First, a forward EEG model was computed using the boundary element method implemented in OpenMEEG [22]. We used the MNI/ICBM152 brain template of Brainstorm as the MRI anatomy [23]. Then, a source model of 15,002 current dipoles was obtained with the inverse sLORETA method implemented in Brainstorm [24]. Dipole orientations were constrained from normal to cortex, and the identity matrix was used as the noise covariance matrix. The EEG preprocessing process rejected a considerable amount of EEG signals, so finally a unique epoch consisting of the first 120 clean seconds of each recording was used. As a result of the source modelling process, a matrix of 15,002 (sources) × 60,000 (time samples) was obtained for each subject.

### 2.3. Fractal Dimension Computation

A 4D fractal dimension (4DFD) approach was used for measuring the spatio-temporal complexity of brain activations. We used the well-known box-counting algorithm [25] to estimate the 4DFD value of brain activations. By using the box-counting algorithm, the fractal dimension (FD) of a set *S* $\in {\mathbb{R}}^{d}$ can be computed as:

(1) $$\mathrm{FD}(S)=\frac{\log\left(n\left(r\right)\right)}{\log\left(\frac{1}{r}\right)}$$

where *n*(*r*) is the number of boxes of scale *r^d^* covering the set *S*. If *S* is not an ideal fractal, then the FD is estimated as the slope of the linear regression of log(*n*(*r*)) vs. log(1/*r*).

**Figure 1:**
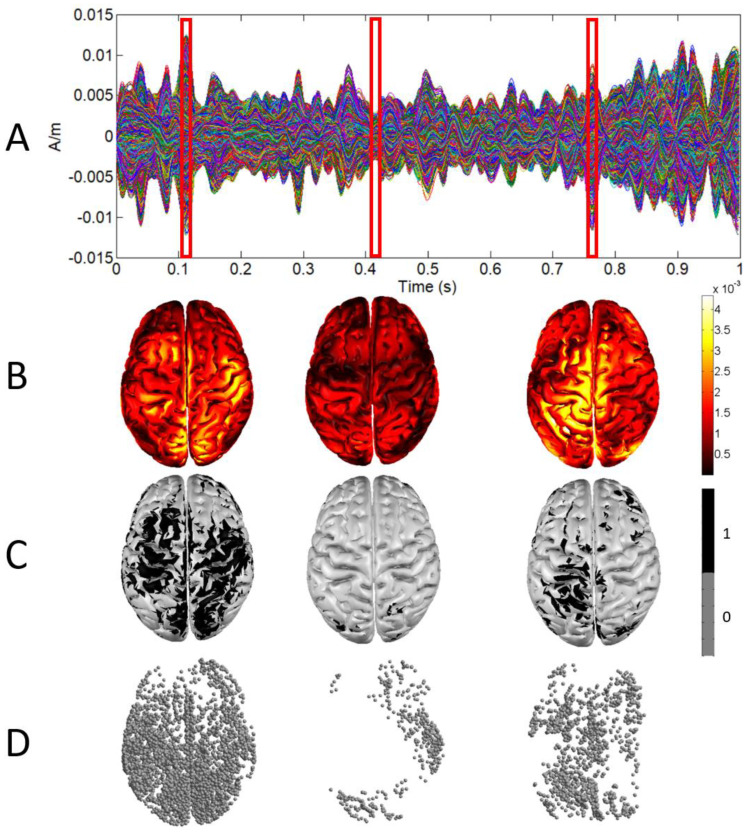
(**A**) Sources activity for one second. (**B**) 3D representation of sources activity at three different samples (red boxes). (**C**) 3D representation after binarization. (**D**) Point cloud defined by sources after binarization.

In our study, the set *S* is composed of the 4D points defined by the 3D locations of cortical activations at each time sample (time is the fourth dimension). In order to obtain this 4D set, firstly, a binary 3D representation of the cortical activations at each time sample was obtained. As an example, Figure 1 shows this binarization process for three samples in the first second of one of the EEG recordings. A source was considered a cortical activation at a time sample if its absolute value was greater than the mean plus the standard deviation of the absolute values of that current for all time samples in the epoch (see Figure 1C,D). This threshold allowed us to select as cortical activations at a time sample those sources having a value significantly greater than the average in the epoch. Moreover, a sufficient number of brain activations were selected in this way, which guarantees a correct estimation of the 4D fractal dimension value obtained through the box-counting algorithm.

**Figure 2:**
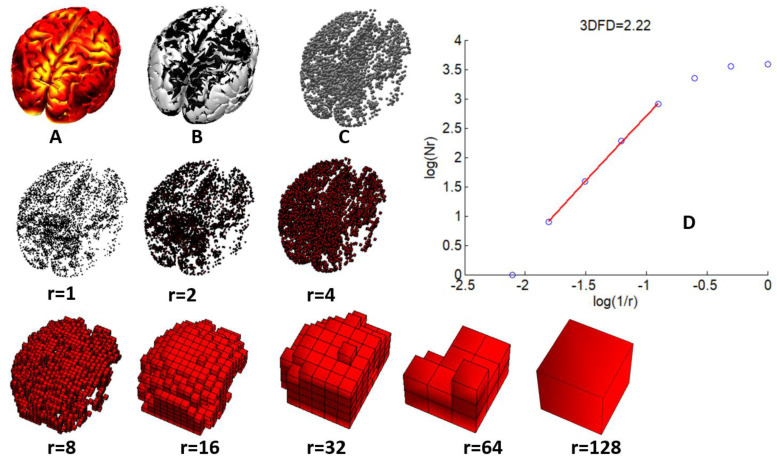
(**A**) Sources activity at a time sample. (**B**) Sources after binarization. (**C**) Point cloud defined by sources binarization. (**D**) 3DFD computation through the box-counting algorithm: log-log plot of number of boxes (N(r)) vs size (1/r) for voxelizations of sizes from r = 1 to r = 128. 3DFD computed as the slope of the regression line for box sizes from r = 8 to r = 64.

Once the 3D point clouds for all the time samples in the 120 s epoch had been computed, the epoch was divided using a sliding window of 1 s without overlapping. Then, the box-counting algorithm was used to obtain the 4DFD value for the 4D set defined in each window by putting together the 3D point clouds of the time samples for the corresponding second. In this way, a total of 120 4DFD values were obtained for each epoch. Figure 2 shows an example of the process of computing the FD of the brain activations using the box-counting algorithm. For the sake of clarity, the example shown in Figure 2 is presented in three dimensions. For the 4D case, the algorithm performed similarly with the same parameters, but having as input the 4D point clouds defined by all the 3D point clouds contained in one second.

Computing the box-counting algorithm in 4D is a highly time-consuming task, so we used the CUDA/C++ source code of the GPU-optimized parallel version of the algorithm provided in [26]. Binarizing brain activations and obtaining the 3D point cloud representation from Brainstorm matrices containing source modelling were computed using home-made MATLAB scripts.

### 2.4. Statistical Analysis

Demographic variables were compared between groups using the nonparametric Mann–Whitney *U* test [27] (age and years of school) and Chi-squared test [28] (sex). In order to study differences in mean values of 4DFD and distributions of 4DFD over time between the PD and HC groups, nonparametric Mann–Whitney *U* tests were performed. Correlations between 4DFD and neuropsychological scores in the PD group were assessed using the nonparametric Spearman’s rank correlation test (ρ) [29]. Since several variables were compared simultaneously, the Spearman’s correlation test was configured using a Bonferroni post-hoc correction for multiple comparisons. In order to measure the performance of 4DFD as a classifier, we used a receiver operating characteristic (ROC) curve analysis [30]. In this analysis, the area under the ROC curve (AUC) was used to measure the classification accuracy of 4DFD.

Results of statistical tests were considered significant when the *p*-value obtained was below 0.05. All statistical analyses were performed in IBM SPSS 28.

## 3. Results

### 3.1. Demographic Results

The demographic and baseline clinical information are summarized in Table 1. Mann–Whitney and Chi-squared tests comparing demographic variables found no significant differences between the PD and HC groups in sex, age, and years of school (see Table 1). These results confirmed that both groups were matched regarding sex, age, and education.

### 3.2. 4DFD Comparison between HC and PD

**Figure 3:**
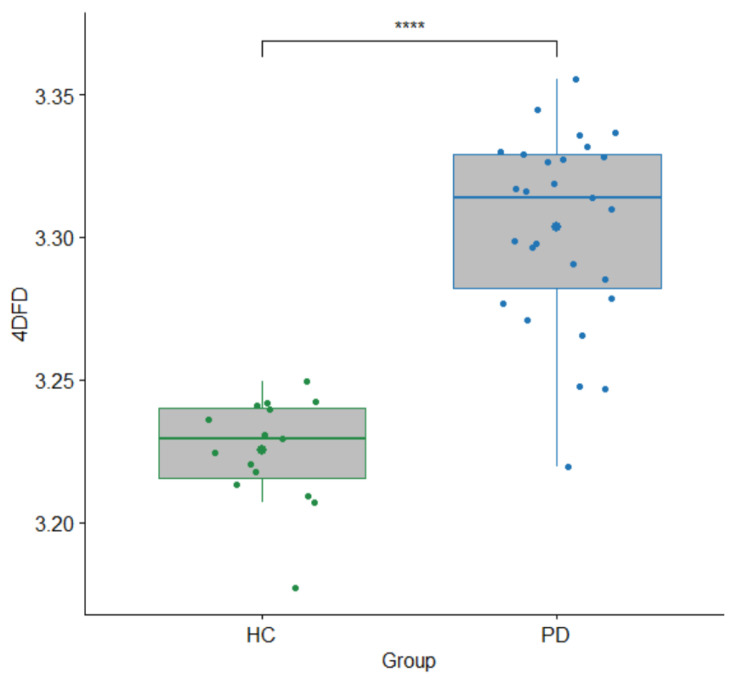
Boxplot with differences in 4DFD between the HC and PD groups. Each 4DFD value corresponds to the average of the 120 4DFD values computed for each subject, one 4DFD for each second in the epoch. *p*-value for Mann–Whitney *U* test (****: *p* < 0.001).

Comparison between HC and PD groups based on 4DFD is shown in Figure 3. For each subject, the mean of the 120 4DFD values computed in the corresponding epoch was considered as a single 4DFD value. Results revealed that 4DFD was significantly higher in the PD group (*U* = 393, *p* < 0.001).

Six patients in the PD group exhibited tremor-related symptoms, so we conducted additional analyses in order to check whether the 4DFD increase in the PD group could be affected by this factor. Our analyses revealed that there was no significant difference in 4DFD values between PD patients with tremor and those without (*U* = 87, *p* = 0.239). Moreover, significant differences in 4DFD values were also found when comparing healthy subjects and PD patients without tremor (*U* = 303, *p* < 0.001).

**Figure 4:**
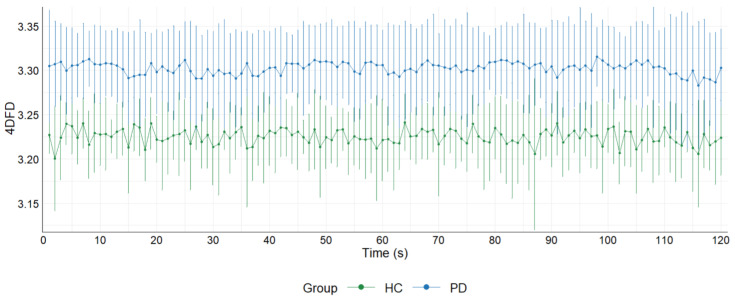
Average 4DFD evolution in 120 s for the PD and HC groups. Each point shows the average value of all the 4DFD computed for that second in the corresponding group. Error bars with standard deviation for each point are also shown.

We also compared the evolution of the 4DFD for the 120 s analyzed in each epoch (see Figure 4). Each point in Figure 4 represents the average of the 4DFD values for all the subjects of the group in the corresponding second. Each point is accompanied by the error bars showing the standard deviation of the mean. Results showed that the distribution of 4DFD values over time is clearly different between groups, with higher values for the PD group (*U* = 14,400, *p* = 0.000).

### 3.3. 4DFD as Classifier for PD

**Figure 5:**
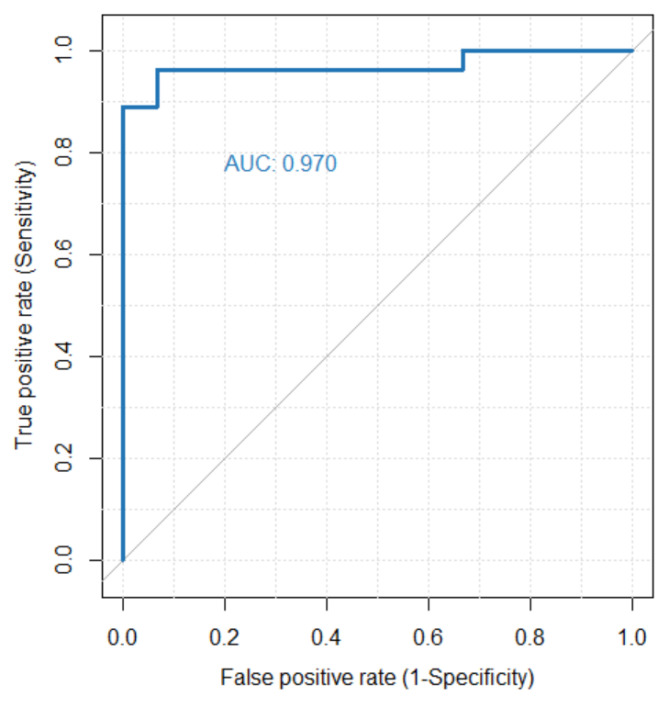
ROC curve analysis evaluating the performance of 4DFD as a classifier for PD. The area under the ROC curve (AUC) is 0.970.

In order to assess the performance of 4DFD as a diagnostic classifier for PD we used an ROC curve analysis (see Figure 5). In that analysis an AUC of 0.970 was achieved, meaning that 4DFD is an excellent classifier.

### 3.4. Correlations between 4DFD and Motor and Neuropsychological Scores

**Figure 6:**
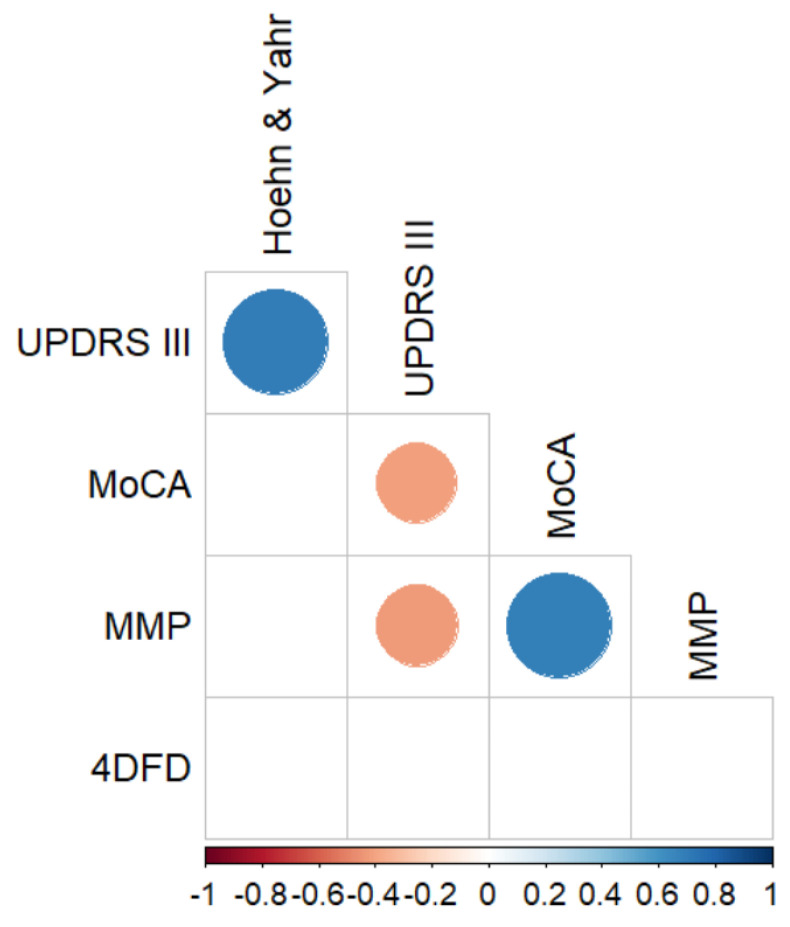
Significant Spearman correlations between neuropsychological scores and 4DFD for the PD group, corrected for multiple comparisons with the Bonferroni post-hoc method. Only statistically significant correlations (*p* < 0.05) are shown. UPDRS III: Unified Parkinson’s Disease Rating Scale part III; MoCA: Montreal Cognitive Assessment; MMP: Mini-Mental Parkinson.

Finally, in order to analyze the relationship between neuropsychological scores and 4DFD in the PD group, we calculated the Spearman correlation coefficient between all these variables with a post-hoc Bonferroni correction for multiple comparisons (see Figure 6). No significant correlations were found between 4DFD and scores of global cognitive impairments (as quantified by MMP and MoCA), but also with motor disability (UPDRS III and Hoehn and Yahr). Additionally, we performed a data reduction of the neuropsychological scores by using a principal component analysis to identify those factors which preserved the maximum variance in the data. The first principal component explained 90.1% of the variance, so we used the score of this first principal component for each PD subject as a new variable to compare. However, the Spearman correlation coefficient between the scores of the first principal component and the 4DFD measure was not significant either (ρ = −0.118, *p* = 0.55).

## 4. Discussion and Conclusions

In the last few years, several studies have proposed complexity measures of EEG as potential neurophysiological markers of early neurodegeneration in PD. These complexity measures were mainly related to the concept of entropy [11,12,13,14,31,32,33]. In the present paper we have also studied the complexity of EEG in PD, but our approach was based on analyzing the fractal structure of the brain activity evolution over time through the 4DFD, a measure which is sensitive to the dispersion of cortical activations.

We found that 4DFD was significantly higher in the PD group compared to the HC (see Figure 3 and Figure 4), suggesting that the brain activity in patients suffering from PD presents a distribution over time more complex than in healthy subjects. Hence, with this study we verified that brain dynamics are modified in PD without dementia, and that the related brain activations over time occupy the 4D space in a more complex way than for the case of healthy subjects. A similar pattern of higher complexity in PD compared to healthy subjects has been observed in the majority of prior studies which analyzed complexity in terms of several different measures of entropy computed directly from the EEG signal [11,12,13,14,33]. This finding of increased complexity has been interpreted as reflecting a state of decreased organization which in turn leads to a decrease in information flow as a result of early alterations in cortical functioning and information processing.

Nevertheless, our approach based on the fractal dimension adds a spatial component which is not taken into account in those previous studies based on entropy. Roughly speaking, the higher the fractal dimension of a shape is, the more complex the object covers the space in which it is defined. In our study, the shape being analyzed was the 4D point cloud defined by the cortical brain activations over time. That means that the dynamics of brain activations in PD patients are distributed in a more complex (meaning more compact, complete, or widespread) way throughout the brain over time than for healthy controls. A possible explanation for this finding is that PD patients show an early impairment in their ability to regulate the flow of information and to allocate attention to filtering stimuli [34]. Therefore, an impairment of attentional processing may imply a more diffuse and changing distribution of brain activations in PD, as revealed by 4DFD. This increase in complexity may also reflect early compensatory mechanisms employed at the cortical level to maintain functionality despite the loss of dopamine-producing neurons. Additionally, the increase in fractal dimension could also be related to the presence of abnormal oscillatory patterns and altered network dynamics observed in Parkinson’s patients. Therefore, further studies on complexity analysis of the specific brain regions associated with functional networks are needed. Two different strategies could be adopted: (1) following a similar 4DFD approach but applied only in those brain regions most affected by PD [35]; and (2) analyzing directly the functional networks using measures of fractal dimension of complex networks [36]. Our future efforts will focus on these directions and try to integrate neurophysiological with additional data types and neuroimaging findings. Moving forward, we recognize the importance of incorporating a broader spectrum of physiological and neuroimaging markers. Alongside our exploration of neurophysiological indicators, we see great potential in integrating cardiac data, such as EKG, into our analysis, and further enriching it with neuroimaging findings. This would provide a more holistic view of the physiological changes associated with PD, possibly unveiling meaningful correlations between the heart, brain, and structural changes as detected by neuroimaging.

There are also some other studies where PD patients presented reduced complexity of EEG compared to control subjects, such as the study of Yi et al. [31] and the one from Keller and colleagues [32]. In the first case, it was found that early-stage PD patients presented a lower value of permutation entropy of resting-state EEG than healthy controls. Similarly, Keller’s study found a decreased complexity of resting-state EEG in the PD group, measured through Tsallis entropy, both compared to healthy subjects at baseline and after three years of cognitive decline. The most recent study that we found in analyzing the complexity of EEG in PD was the one presented by Pappalettera et al. [33]. Again, the results of this study showed that PD patients presented significantly higher values of entropy (approximate entropy) than elderly healthy controls when analyzing resting-state EEG signals. The variability in these findings could be attributed to the relatively few studies that concentrate on the significance of different complexity metrics in predicting early cognitive impairments, as well as the inclusion of PD patients who do not have dementia but already present pre-morbid minimal cognitive impairment (MCI) profiles.

4DFD also performed very well as a classifier (see Figure 5), separating PD and HC groups with very few exceptions. This means that 4DFD could be used as one of the features in advanced deep learning and machine learning methods for detecting PD [37,38].

No significant correlations were found between 4DFD and the severity of motor impairment in the PD group, nor were any correlations found with overall cognitive impairment scores. Thus, based on these findings, the spatio-temporal fractal dimension does not appear to be a suitable descriptor for the progression of motor and cognitive symptoms in PD. We hypothesize that, although 4DFD can detect general changes in brain dynamics between PD patients and healthy subjects, the subtle progressive neurodegeneration in PD may not be adequately captured by this whole-brain complexity measure. This could be attributed to the fact that the patients do not exhibit major cognitive impairments. Targeted follow-up studies could help clarify the role of fractal dimension as a predictor of progression into MCI and dementia.

Regarding previous studies using fractal analysis for assessing the complexity of EEG signal in PD, to the best of our knowledge there is only one study, the one presented by Muller and collaborators [11], based on correlation dimension. This previous study showed the ability of correlation dimension in differentiating PD and HC when subjects were performing motor tasks. However, no significant differences between groups were found for the resting-state EEG signal. Therefore, our approach based on 4DFD adds a new capacity of fractal analysis for differentiating PD from HC from resting-state EEG.

One requirement of the FD computation based on the box-counting algorithm is the necessity of binarizing the signal in order to construct the boxes at different scales [26]. This process of binarization naturally implies an information loss, which could affect more clearly in the case of using resting-state EEG signals instead of processing event or stimulus evoked potentials, where it is easier to select the brain activations based on significant changes in the signal due to the event or stimulus [7]. Therefore, further studies should be conducted using alternative methods for computing the fractal dimension which avoid the binarization of the signal. The differential box-counting algorithm (DBC) [39] is one of these methods, allowing the computation of the fractal dimension of gray-scale images. Our future efforts will focus on adapting the DBC algorithm to be used with the spatio-temporal 4D structures obtained from the resting-state EEG signal. In future research it may also be intriguing to investigate the potential of fractal dimension as a biomarker of minimal cognitive impairment, for example, by monitoring the cognitive progression of the disease and evaluating patients through longitudinal EEG recordings obtained at follow-up.

While our study has provided valuable insights into the complexity of resting-state EEG signals in PD patients, we acknowledge that our analysis of brain dynamics was confined to the resting-state. Future research would benefit from an extended analysis that includes active-state EEG, possibly during specifically designed cognitive tasks. Such an approach could provide insights into brain activity dynamics and complexity in PD and help unravel whether the observed changes in complexity are state-dependent and to what extent they are influenced by engagement in cognitive tasks.

In conclusion, in this study we have presented a novel methodology for performing the complexity analysis of resting-state EEG signals in PD. Our method is based on computing the 4DFD of the brain activations induced from the EEG signal, allowing us to perform in this way a spatio-temporal analysis. This complexity analysis based on the 4DFD of resting-state EEG obtained excellent performance in differentiating PD patients from healthy subjects. Our findings indicate that 4DFD is a promising tool for gaining deeper insights into the early cortical neurodegeneration characteristics of PD and to better plan rehabilitation treatment.

## Figures and Tables

**Table 1:** Study demographics and clinical data. Values expressed as mean ± standard deviation. Education given as years of school. UPDRS III: Unified Parkinson’s Disease Rating Scale part III; MoCA: Montreal Cognitive Assessment; MMP: Mini-Mental Parkinson.

	PD	HC	Test, *p*-Value
N	27	15	
Sex (F:M)	12:15	7:8	χ^2^ = 0.019, *p* = 0.89 ^a^
Age	69.59 ± 6.74	67.53 ± 4.94	*U* = 249.5, *p* = 0.22 ^b^
Education	12.74 ± 3.79	12.80 ± 2.95	*U* = 195.0, *p* = 0.84 ^b^
Disease duration	10.24 ± 6.78		
Hoehn and Yahr	2.41 ± 0.42		
UPDRS III	39.00 ± 9.79		
MoCA	25.01 ± 2.65		
MMP	28.62 ± 2.45		

^a^ Chi-squared test. ^b^ Mann–Whitney test.

## Data Availability

The data presented in this study are available on request to fsaibene@dongnocchi.it. The data are not publicly available due to privacy restrictions.

## References

[1] Mandelbrot B.B. (1983). The Fractal Geometry of Nature.

[2] Ziukelis E.T., Mak E., Dounavi M.E., Su L., O’Brien J.T. (2022). Fractal dimension of the brain in neurodegenerative disease and dementia: A systematic review. Ageing Res. Rev..

[3] Lau Z.J., Pham T., Chen S.H.A., Makowski D. (2022). Brain entropy, fractal dimensions and predictability: A review of complexity measures for EEG in healthy and neuropsychiatric populations. Eur. J. Neurosci..

[4] Grassberger P., Procaccia I. (1983). Measuring the strangeness of strange attractors. Phys. D Nonlinear Phenom..

[5] Higuchi T. (1988). Approach to an irregular time series on the basis of the fractal theory. Phys. D Nonlinear Phenom..

[6] Richman J.S., Moorman J.R. (2000). Physiological time-series analysis using approximate entropy and sample entropy. Am. J. Physiol. Circ. Physiol..

[7] Ruiz de Miras J., Soler F., Iglesias-Parro S., Ibáñez-Molina A.J., Casali A.G., Laureys S., Massimini M., Esteban F.J., Navas J., Langa J.A. (2019). Fractal dimension analysis of states of consciousness and unconsciousness using transcranial magnetic stimulation. Comput. Methods Programs Biomed..

[8] Valls-Solé J., Valldeoriola F. (2002). Neurophysiological correlate of clinical signs in Parkinson’s disease. Clin. Neurophysiol..

[9] Jankovic J. (2008). Parkinson’s disease: Clinical features and diagnosis. J. Neurol. Neurosurg. Psychiatry.

[10] Kalia L.V., Lang A.E. (2015). Parkinson’s disease. Lancet.

[11] Müller V., Lutzenberger W., Pulvermüller F., Mohr B., Birbaumer N. (2001). Investigation of brain dynamics in Parkinson’s disease by methods derived from nonlinear dynamics. Exp. Brain Res..

[12] Pezard L., Jech R., Růžička E. (2001). Investigation of non-linear properties of multichannel EEG in the early stages of Parkinson’s disease. Clin. Neurophysiol..

[13] Chung C.C., Kang J.H., Yuan R.Y., Wu D., Chen C.C., Chi N.F., Chen P.C., Hu C.J. (2013). Multiscale entropy analysis of electroencephalography during sleep in patients with parkinson disease. Clin. EEG Neurosci..

[14] Han C.X., Wang J., Yi G.S., Che Y.Q. (2013). Investigation of EEG abnormalities in the early stage of Parkinson’s disease. Cogn. Neurodyn..

[15] Postuma R.B., Berg D., Stern M., Poewe W., Olanow C.W., Oertel W., Obeso J., Marek K., Litvan I., Lang A.E. (2015). MDS clinical diagnostic criteria for Parkinson’s disease. Mov. Disord..

[16] Boel J.A., de Bie R.M.A., Schmand B.A., Dalrymple-Alford J.C., Marras C., Adler C.H., Goldman J.G., Tröster A.I., Burn D.J., Litvan I. (2022). Level I PD-MCI Using Global Cognitive Tests and the Risk for Parkinson’s Disease Dementia. Mov. Disord. Clin. Pract..

[17] Dubois B., Burn D., Goetz C., Aarsland D., Brown R.G., Broe G.A., Dickson D., Duyckaerts C., Cummings J., Gauthier S. (2007). Diagnostic procedures for Parkinson’s disease dementia: Recommendations from the movement disorder society task force. Mov. Disord..

[18] Goetz C.G., Tilley B.C., Shaftman S.R., Stebbins G.T., Fahn S., Martinez-Martin P., Poewe W., Sampaio C., Stern M.B., Dodel R. (2008). Movement Disorder Society-sponsored revision of the Unified Parkinson’s Disease Rating Scale (MDS-UPDRS): Scale presentation and clinimetric testing results. Mov. Disord..

[19] Hoehn M.M., Yahr M.D. (1967). Parkinsonism. Neurology.

[20] Delorme A., Makeig S. (2004). EEGLAB: An open source toolbox for analysis of single-trial EEG dynamics including independent component analysis. J. Neurosci. Methods.

[21] Tadel F., Baillet S., Mosher J.C., Pantazis D., Leahy R.M. (2011). Brainstorm: A user-friendly application for MEG/EEG analysis. Comput. Intell. Neurosci..

[22] Gramfort A., Papadopoulo T., Olivi E., Clerc M. (2010). OpenMEEG: Opensource software for quasistatic bioelectromagnetics. Biomed. Eng. Online.

[23] Fonov V., Evans A., McKinstry R., Almli C., Collins D. (2009). Unbiased nonlinear average age-appropriate brain templates from birth to adulthood. Neuroimage.

[24] Pascual-Marqui R.D. (2002). Standardized low-resolution brain electromagnetic tomography (sLORETA): Technical details. Methods Find. Exp. Clin. Pharmacol..

[25] Russell D.A., Hanson J.D., Ott E. (1980). Dimension of strange attractors. Phys. Rev. Lett..

[26] Ruiz de Miras J., Posadas M.A., Ibáñez-Molina A.J., Soriano M.F., Iglesias-Parro S. (2023). Fast computation of fractal dimension for 2D, 3D and 4D data. J. Comput. Sci..

[27] Mann H.B., Whitney D.R. (1947). On a Test of Whether one of Two Random Variables is Stochastically Larger than the Other. Ann. Math. Stat..

[28] Pearson K.X. (1900). On the criterion that a given system of deviations from the probable in the case of a correlated system of variables is such that it can be reasonably supposed to have arisen from random sampling. Lond. Edinb. Dublin Philos. Mag. J. Sci..

[29] Spearman C. (1904). The Proof and Measurement of Association between Two Things. Am. J. Psychol..

[30] Hanley J.A., McNeil B.J. (1982). The meaning and use of the area under a receiver operating characteristic (ROC) curve. Radiology.

[31] Yi G.S., Wang J., Deng B., Wei X. (2017). Le Complexity of resting-state EEG activity in the patients with early-stage Parkinson’s disease. Cogn. Neurodyn..

[32] Keller S.M., Gschwandtner U., Meyer A., Chaturvedi M., Roth V., Fuhr P. (2020). Cognitive decline in Parkinson’s disease is associated with reduced complexity of EEG at baseline. Brain Commun..

[33] Pappalettera C., Miraglia F., Cotelli M., Rossini P.M., Vecchio F. (2022). Analysis of complexity in the EEG activity of Parkinson’s disease patients by means of approximate entropy. GeroScience.

[34] Watson G.S., Leverenz J.B. (2010). Profile of Cognitive Impairment in Parkinson’s Disease. Brain Pathol..

[35] Leviashvili S., Ezra Y., Droby A., Ding H., Groppa S., Mirelman A., Muthuraman M., Maidan I. (2022). EEG-Based Mapping of Resting-State Functional Brain Networks in Patients with Parkinson’s Disease. Biomimetics.

[36] Nahli F., Paramonov A., Soliman N.F., Aleisa H.N., Alkanhel R., Muthanna A., Ateya A.A. (2022). Novel Path Counting-Based Method for Fractal Dimension Estimation of the Ultra-Dense Networks. Intell. Autom. Soft Comput..

[37] Aljalal M., Aldosari S.A., Molinas M., AlSharabi K., Alturki F.A. (2022). Detection of Parkinson’s disease from EEG signals using discrete wavelet transform, different entropy measures, and machine learning techniques. Sci. Rep..

[38] Zhang R., Jia J., Zhang R. (2022). EEG analysis of Parkinson’s disease using time–frequency analysis and deep learning. Biomed. Signal Process. Control.

[39] Jiang W., Liu Y., Wang J., Li R., Liu X., Zhang J. (2022). Problems of the Grid Size Selection in Differential Box-Counting (DBC) Methods and an Improvement Strategy. Entropy.

